# Biological Characterization and Inhibition of *Streptococcus pyogenes* ZUH1 Causing Chronic Cystitis by *Crocus sativus* Methanol Extract, Bee Honey Alone or in Combination with Antibiotics: An In Vitro Study

**DOI:** 10.3390/molecules24162903

**Published:** 2019-08-09

**Authors:** Seham Abdel-Shafi, Abdul-Raouf Al-Mohammadi, Sara Hamdi, Ahmed H. Moustafa, Gamal Enan

**Affiliations:** 1 Department of Botany and Microbiology, Faculty of Science, Zagazig University, Zagazig 44519, Egypt; 2 Department of Science, King Khalid Military Academy, P.O. Box 22140, Riyadh 11495, Saudi Arabia; 3 Department of Chemistry, Faculty of Science, Zagazig University, Zagazig 44519, Egypt

**Keywords:** Group A streptococci (GAS), *S. pyogenes* ZUH1, virulence factors, *Crocus sativus*, CSME, GC-MS, IR analysis, transmission electron microscopy (TEM)

## Abstract

*Streptococcus pyogenes* (*S. pyogenes*) ZUH1 was isolated and characterized using morphological, cultural and biochemical methods. The results showed that the marker genes (namely spyCEP, ssa, sic, sdaB and speG) indicating group A streptococci (GAS) were detected in the *S. pyogenes* genome. The results showed that the *S. pyogenes* strain was inhibited by *Crocus sativus* methanol extract (CSME), bee honey (BH) and catfish glycoprotein (CFG). The inhibitory activity of these natural agents were compared with standard antibiotics such as Ceftazidime (30 μg/mL), Cefoperazone (75 μg/mL), Cefoxitin (30 μg/mL) and Imipenem (10 μg/mL). There was a synergistic effect between certain antibiotics and CSME. GC-MS and IR analysis of CSME showed different cyclic ketones, aldehydes, esters, alcohols and acids. The main compounds were tetradecanoic acid, safranal and isophorone. Transmission electron microscopy (TEM) images of *S. pyogenes* cells treated with CSME showed signs of an irregular wrinkled outer surface, fragmentation, adhesion and aggregation of damaged bacterial cells or cellular debris. The marker genes (spyCEP, ssa, sic, sdaB and speG) could be used as a rapid diagnostic tool for GAS. CSME, BH and CFG showed distinctive anti-streptococcal activity either alone or in combinations with antibiotics; their action on *S. pyogenes* cells was studied by TEM. There was a synergistic effect between antibiotics and *Crocus sativus*, bee honey, and glycoprotein against* S. pyogenes* ZUH1. The action of natural agents on the pathogenic cells was shown using TEM.

## 1. Introduction

Herbal extracts are currently used as drugs either alone or in combination with antibiotics for possible use as alternative antibacterial agents for the inhibition of antibiotic resistant bacteria [1]. Group A streptococci (GAS) have been deduced by Lancefield classification and include bacteria belonging to the pathogenic organism *S. pyogenes*; such bacteria are associated with a diverse group of human diseases including suppurative (pus forming) pharyngitis, tonsillitis, puerperal fever and cutaneous wound infections. Many acute non-suppurative infections are caused by GAS such as acute glomerulonephritis, skin erythema and (indirectly) rheumatic fever [2,3].

Recent studies have shown that certain strains of GAS are multi-drug resistant [3]. Due to such phenomena and their survival at low temperatures in pus and sputum, the search for other alternative antibacterial agents is necessary. The pathogenic properties of GAS strains are often linked to the production of virulence factors such as toxins, proteases or DNases. Toxins can be a predictor of GAS strain invasiveness [2,3].

Virulence factors are not equally distributed among GAS. Some of them are chromosomally encoded while others are related to the presence of mobile genetic elements. The detection of GAS virulence factors is necessary for strain characterization and their presence/absence can be used as a diagnostic method and simple tool in clinical diagnosis. The severity of GAS infections depends on the presence of virulence factors [4]. There is a need to search for potential novel drugs which might be helpful for the treatment of bacterial infections in general and GAS infections in particular [5,6,7]. Phytochemical substances can be used in the treatment of resistant microbes. Their use in combination with antibiotics against resistant bacteria leads to new choices for the treatment of microbial diseases [8,9,10,11].

*Crocus sativus* flowers contain bioactive compounds with antimicrobial properties against oral pathogens, particularly *Streptococci*. These compounds have been used to prepare mouthwashes and antimicrobial drugs [1]. *C. sativus* extract showed both antioxidant and antimicrobial activities due to its bioactive compounds such as flavonoids, gallic acid, pyrogallol and other phenols [12]. GC-MS analysis of *C. sativus* showed that it contains safranal, isophorone and 4-ketoisophorone [13,14].* C. sativus* contains more than 150 volatile aroma compounds. Safranal, crocin and picrocrocin are the main compounds. It has potential antibacterial activity against *S. aureus.* Safranal (C_10_H_14_O) is one of the main components of saffron essential oil. It is responsible for the aroma of saffron. It prevents stomach ulcers and it also contains antimicrobial and antitumor agents [13,15]. Therefore, studies regarding the use of *C. sativus* extracts as an antimicrobial agent should be extended to use the extract in different proportions with antibiotics in combination to provide a broader antimicrobial spectrum; the present manuscript tried to fill this gap.

Work was undertaken to characterize certain GAS pathogens by both biochemical and molecular techniques. The challenge was then to inhibit a GAS pathogen (*S. pyogenes*) using natural agents such as *Crocus sativus* methanol extract (CSME), bee honey (BH) and catfish glycoprotein (CFG). The antibacterial activity of the combination of antibiotics and bioactive compounds elucidated by GC-MS analysis of CSME was tested; the action of the natural agents tested herein on *S. pyogenes* was further investigated by transmission electron microscopy (TEM). 

## 2. Materials and Methods

### 2.1. Isolation and Characterization of the ZUH1 Strain

A urine swab was taken from a 60-year-old man suffering from cystitis and admitted to Zagazig University Hospital (ZUH), Zagazig, Egypt. It was streaked onto Brain Heart Infusion agar (BHI, Oxoid). After incubation at 35 °C for 48 h, a pure single colony with a yellowish white appearance was picked up by a sterile needle and streaked onto a slope culture of the same medium that was incubated at 35 °C for 48 h. This strain was designated ZUH1 and was used for further work. 

For characterization of the ZUH1 strain, a Gram stain was carried out; it showed Gram positive coccoid and catalase negative cells. Blood hemolysis was carried out as described previously [16]. An anti-streptolysin O assay (ASO) was carried out as reported previously [17]. A lipase test was carried out using olive oil medium as described previously [18]. Phospholipase and protease activities were determined as described previously [19,20]. Further biochemical identification testes were carried out using API-Streptococci Kits (Biomereux, France).

### 2.2. Molecular Identification of the ZUH1 Strain

DNA was isolated from the ZUH1 strain using the method adopted previously [21]. The primers used for the molecular detection of Group A streptococci (GAS) were provided from Promega Co. (Table 1). Polymerase chain reaction (PCR) was carried out using the primers for the spyCEP, ssa, sic, sdaB and speG genes (markers for GAS (*S. pyogenes*)). spyCEP encodes for protease activity, which digests the interleukin (IL)-8 protein and results in a significant decrease in host phagocytic cell activity. The other genes (ssa, sic, sdaB and speG) encode superantigens that identify GAS. The PCR rounds were carried out after mixing DNA preparations with the primers given in Table 1, according to the method published previously [4].

### 2.3. Antibiotic Susceptibility Test

The twelve antibiotics used in this study were obtained from High-Lab Company (Zagazig City, Egypt) and are given in Table 2. These antibiotics were used to carry out the antimicrobial susceptibility test. The antibiotic sensitivity test was carried out via a disc diffusion assay in which antibiotic-impregnated wafers were used to determine the sensitivity of the ZUH1 strain. The antibiotic discs were placed onto the surface of BHI agar (Oxoid) that was previously seeded with a suspension of the ZUH1 strain. The disks were laid on the BHI agar surface with appropriate distances separating them from each other. The plates were incubated at 37 °C for 24–48 h and the inhibition zone diameter (IZD) (mm) was measured using a millimeter ruler. The diameters of the antibiotic discs were subtracted from the total zone diameters. Results were calculated according to the rules of the Clinical and Laboratory Standards Institute [22].

### 2.4. Preparation of C. sativus Extracts

*C. sativus* flowers were air dried first at room temperature and then in an oven at 45 °C to constant weights. Powders of these plants were prepared by grinding in a Bench Grinder (Molinex, Egypt) and they were subsequently kept away from moisture in a closed plastic container. About 10 g of powdered plant material was soaked separately in either 100 mL distilled water or in an organic solvent (methanol, 95% ethanol, petroleum ether, acetone, or benzene) for 72 h. Each mixture was stirred using a sterile glass rod. After extraction, each extract was passed through Whatman No.1 filter paper. The obtained filtrate was reduced to dryness by removing the solvents under air at atmospheric pressure. Each dried crude extract was dissolved in 2 mL distilled water and was sterilized by filtration (0.45 µm, Milipore), and then stored in Eppendorf tubes at 5 °C until antimicrobial activity tests were performed [23].

### 2.5. Preparation of BH Solutions and CFG

To compare the results obtained from CSME, both BH and CFG were also assessed for their anti-streptococcal activity. The bee honey sample was gathered and provided by a bee-keeper from the Kafr-Sakr area, Egypt. This BH sample was aseptically collected in a sterile screw capped bottle (100 mL) and kept in a dry place at room temperature overnight before being transported to the laboratory. BH solutions were prepared immediately prior their testing by diluting honey to the required concentrations (10%, 20%, 30%, 40%, 50%, 60%, 70%, 80% and 90% *v*/*v*) [24].

CFG samples were prepared as described previously [25]. Different concentrations of this CFG (10 µg/mL, 25 µg/mL, 50 µg/mL,100 µg/mL, 250 µg/mL and 500 µg/mL) were prepared and kept at 4 °C in a refrigerator until used.

### 2.6. Bioassay of Inhibitory Activity of Plant Extracts, BH and CFG

Sterilized filter paper discs (6 mm diameter) were prepared and the concentrations of the natural agents listed in Table 3 were added by automatic pipette to these filter paper discs under aseptic conditions. These discs were then placed onto BHI agar plates that were seeded previously with a cell suspension of log phase *S. pyogenes* cells. The controls were filter paper discs soaked in sterile deionized water. Samples and controls were incubated at 37 °C for 24–48 h. IZD was measured after subtracting the diameter of the paper disc [26].

### 2.7. Minimum Inhibitory Concentration (MIC) of C. sativus Methanol Extract (CSME)

*C. sativus* extracts obtained with the use of various organic solvents were initially tested. Serial dilutions of CSME were made in sterile deionized water and DMSO at a ratio of 1:1 (*v*/*v*). Then, sterile filter paper discs were saturated with the CSME dilutions and antibacterial activity was studied by disc diffusion assay as described above. MIC was visually identified as the lowest concentration of CSME that inhibited visible growth.

### 2.8. Anti-Streptococcal Activity of the Combination of Antibiotics and Either CSME or BH

The antibiotics listed in Table 2 that inhibited the *S. pyogenes* strain were mixed with the MIC values of either CSME or BH. Sterile filter paper discs were impregnated by these combinations and assayed for their anti-streptococcal activity as described above. In addition, different concentrations of the antibiotic imipenem (the most effective antibiotic) and either CSME or BH were tested for their anti-streptococcal activity. Mixtures of either the natural agent (CSME or BH) and the antibiotic imipenem were made as follows: 80 µg/mL natural agent + 20 µg/mL imipenem, 60 µg/mL natural agent + 40 µg/mL imipenem, 40 µg/mL natural agent + 60 µg/mL imipenem, and 20 µg/mL natural agent + 80 µg/mL imipenem. Filter paper discs of 6 mm diameter were soaked in each combination and the experiment was carried out as described above.

### 2.9. Instrumental Analysis of CSME

CSME was prepared as described above. For gas chromatography-mass spectrometry (GC-MS) analysis, 3µL of CSME was injected into the GC-MS equipment (Trace GC 1310-ISQ Mass Spectrometer, Thermo Scientific, Austin, TX, USA) with a direct capillary column TG–5MS (30 m × 0.25 mm × 0.25 µm film thickness). The column oven temperature was initially held at 50 °C and then increased by 5 °C/min to 250 °C, held for 2 min, and then increased to 300 °C by 15 °C/min. The injector temperature was kept at 250 °C. Helium was used as a carrier gas at a constant flow rate of 1 mL/min. The solvent delay was 3 min and diluted samples of 3 µL were injected automatically using an Autosampler AS3000 coupled with GC in the splitless mode. Mass spectra were collected at 70 eV ionization voltages over the range of *m*/*z* 50–550 in full scan mode. The ion source and transfer line temperatures were set at 200°C and 270 °C, respectively. The components were identified by comparison of their retention times and mass spectra with those of the WILEY 09 and NIST 11 mass spectral databases. For Infra Red (IR) spectra was carried out by yang apparatus 2006.

### 2.10. Transmission Electron Microscopy (TEM) of S. pyogenes ZUH1

CSME (20 µg/mL), BH (20 µg/mL) and imipenem (10 µg/mL) were chosen for TEM investigations. Also, 50 µg/mL and 500 µg/mL of CFG were chosen for TEM investigations. BHI broths were inoculated with *S. pyogenes* and incubated at 37 °C for 24 h. Cell suspensions of the growing cells were subsequently diluted to 1 × 10^5^ CFU/mL using peptone buffer solution (0.1% peptone plus 0.85% NaCl), treated with the above-mentioned concentrations of natural agents used and incubated for 4 h at 37 °C.

Bacterial cells were fixed in glutaraldehyde (2.5% in 0.1 M phosphate buffer, pH 7.4) for 1 h, rinsed for 10 min with 0.1 M phosphate buffer (pH 7.4) and post-fixed with 1% osmium tetraoxide for 2 h at 4 °C. The washing step was repeated and the cells were dehydrated sequentially using 30%, 50%, 70% and 95% acetone for 15 min each and finally with 100% acetone three times for 30 min. Subsequently, cells were treated with propylene oxide twice for 10 min at 4 °C and sequentially filtrated with a mixture of propylene oxide and Durcupan’s ACM epoxy resin (3:1, 1:1 and 1:3) for 45 min. Polymerization of the resin to form specimen blocks was performed in an oven at 60 °C for 72 h. The specimen blocks were sectioned with a diamond knife in a Reichert Ultracut R ultramicrotome (Leica, Wetzler, Germany). Thin sections (70–80 nm) were placed on 300 mesh copper grids, stained for 15–20 min in uranyl:ethyl alcohol (1:1) and then washed three times with saline solution for 2 min. A drop of Reynol’s lead citrate was added before examination using a TEM (JEOL, Japan).

### 2.11. Ethical Approval

This study was approved by the institutional review board, Faculty of Science, Zagazig University, Egypt.

## 3. Results

### 3.1. Biochemical and Molecular Characterization of S. pyogenes ZUH1

The ZUH1 (Zagazig University Hospital, Egypt) isolate showed Gram positive cocci and a negative catalase reaction. API streptococci kits were used for studying the biochemical tests carried out on this isolate according to the manufacturer’s instructions (Biomereux, France). They showed that the ZUH1 strain isolated from a 60-year-old man suffering from cystitis was *S. pyogenes* and we designated it as *S. pyogenes* ZUH1 (*S. pyogenes*).

**Figure 1:**
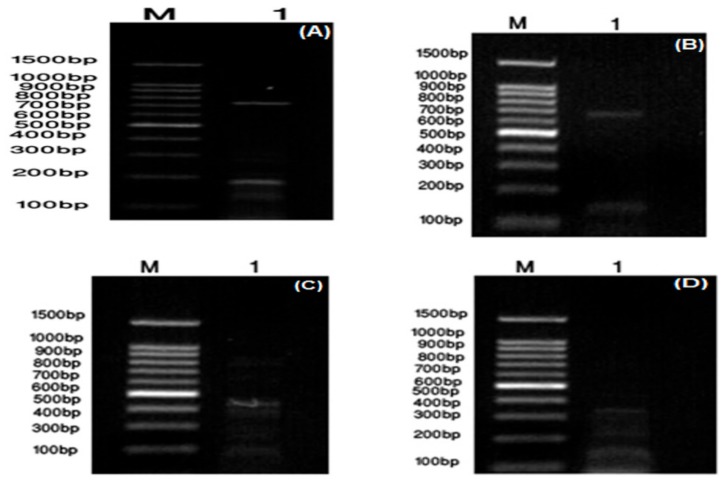
Detection of *S. pyogenes* ZUH1 virulence factors in four multiplex PCR reactions (**A**): spyCEP (786 bp); (**B**): ssa (678 bp) and sic (150 bp); (**C**): sdaB (440 bp); (**D**): speG (384 bp).

Further diagnostic and identification tests were carried out. Anti-streptolysin O (ASO) was positive and its agglutination titer was 200 IU/mL (Supplementary Figure S1A). *S. pyogenes* ZUH1 showed β-hemolytic activity in blood agar (Supplementary Figure S1B) and positive lipase activity (Supplementary Figure S1C). It showed positive phospholipase protease activity as clear zones appeared around bacterial streaks in media supplemented with egg yolk and casein (Supplementary Figure S1D,E, respectively). Some specific marker genes for GAS were detected by PCR reaction within the *S. pyogenes* ZUH1 genome using the specific primers given in Table 1. Certain virulence genes were detected (spyCEP, ssa plus sic, sdaB and speG) as DNA bands of about 786 bp, 678 bp, 150 bp, 440 bp and 384 bp respectively were clearly seen (Figure 1).

### 3.2. Antibiotic Susceptibility of S. pyogenes

Twelve antibiotics (tetracycline, piperacillin, clindamycin, penicillin G, ceftazidime, cefoxitin, cefoperazone, imipenem, amikacin, ampicillin, aztreonam, and norfloxacin) were used to carry out the antimicrobial susceptibility test. The results given in Table 2 showed that imipenem and bacitracin were the most potent antibiotics (36 mm IZD). The experimental *S. pyogenes* strain was resistant to ampicillin, aztreonam and norfloxacin. The other antibiotics tested showed moderate anti-streptococcal activity (20–26 mm IZD).

### 3.3. Inhibition of S. Pyogenes by C. Sativus Extracts, BH and CFG

The inhibition of *S. pyogenes* by *C. sativus* solvent extracts was studied by disc diffusion assay. It was shown that methanol was the most effective solvent for the extraction of *C. sativus* bioactive compounds as an IZD of about 35 mm was shown against *S. pyogenes* (Supplementary Figure S2). The solvents used can be arranged in the following descending order according to their extractability of the bioactive molecules from the *C. sativus* plant: methanol > acetone > petroleum ether > benzene > ethanol. To compare the inhibition profiles of *S. pyogenes* obtained by CSME with other natural agents such as BH and CFG, many dilutions of either BH or CFG were made (*v*/*v*) (Table 3) using sterile distilled water and DMSO at 1:1 (*v*/*v*). Crude honey (100%) showed the highest anti-streptococcal activity. By decreasing honey concentrations (using more diluted honey), the diameter of inhibition zones was decreased (Table 3 and Supplementary Figure S3). Also, it was shown that 500 µg/mL CFG was the most inhibitory concentration. When the concentration of CFG was decreased, anti-streptococcal activity was in turn decreased (Supplementary Figure S4). The MIC values of CSME, BH and CFG were 20 µg/mL, 20 g/100 mL and 50 µg/mL, respectively (Table 3).

### 3.4. Anti-Streptococcal Effect of Combinations of Different Antibiotics and Either CSME or BH

**Figure 2:**
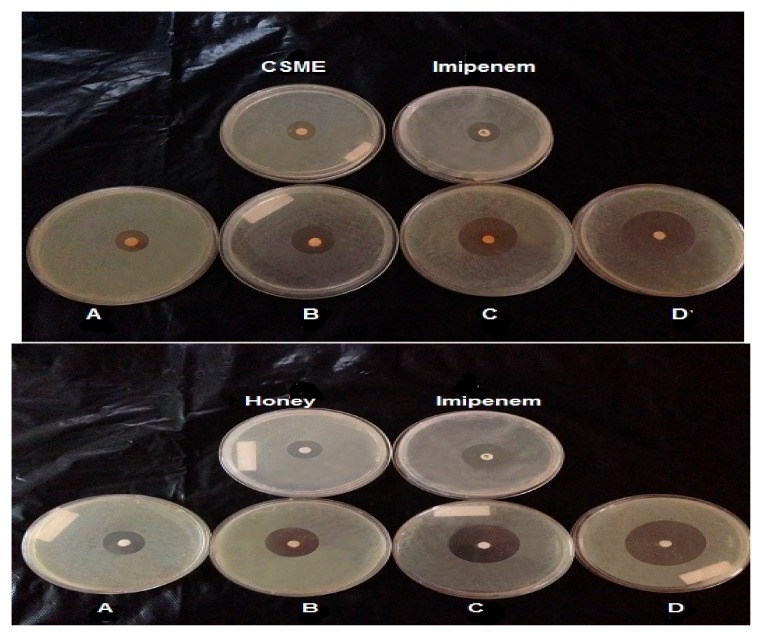
Synergistic effect of the natural product and imipenem combination by disc diffusion assay on *S. pyogenes* ZUH1. (**A**): 20 μg/mL (CSME or BH) + 80 μg/mL imipenem, (**B**): 40 μg/mL natural substance + 60 μg/mL imipenem, (**C**): 60 μg/mL natural substance + 40 μg/mL imipenem, (**D**): 80 μg/mL natural substance + 20 μg/mL imipenem. In the first picture (up picture) the natural product is CSME + imipenem in A–D; in the second picture (down picture) the natural product is Honey+ imipenem in A–D.

To check whether combinations of antibiotics with either BH or CSME have positive synergistic effects and in turn wider anti-streptococcal activity, the antibiotics with the maximal antibacterial activity (ceftazidime, cefoxitin, cefoperazone and imipenem) at their concentrations given in Table 4 and Figure 2 were mixed with the MIC concentrations of either BH (20 g/100 mL) or CSME (20 μg/mL). Distinctive anti-streptococcal activity was obtained from combinations of antibiotics with either BH or CSME. The inhibition zones were higher than that obtained by antibiotics alone (Table 4 and Figure 2).

### 3.5. GC-MS and IR Analysis of CSME

**Figure 3:**
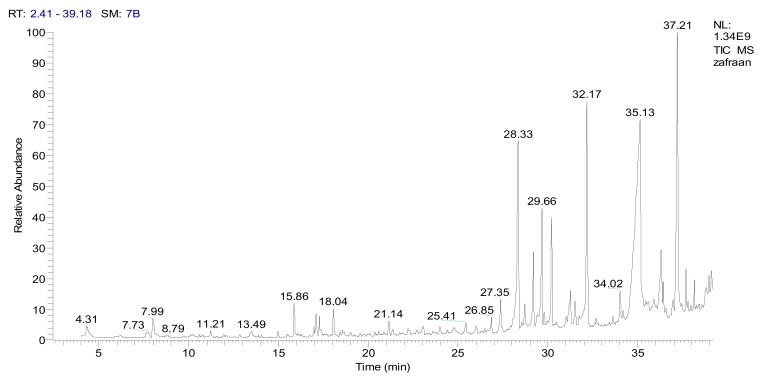
The TIC chromatogram of CSME using GC-MS. RT- Retention Time; SM- Signal in Method; NL-Noise Level.

**Figure 4:**
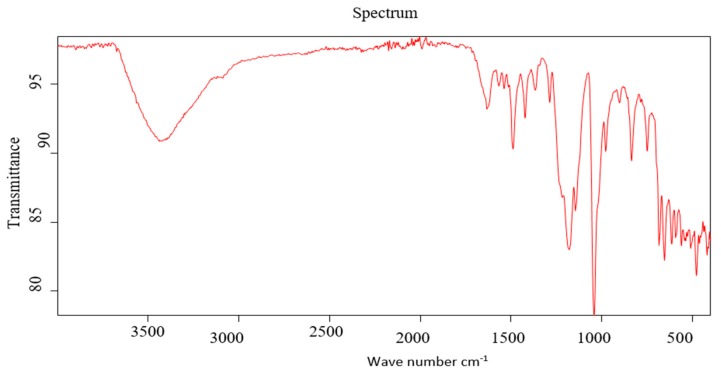
IR of CSME.

In the current study, CSME was subjected to GC-MS and IR analysis to detect the functional groups in the extracted compounds. GC-MS showed eight principal peaks that correspond to eight active compounds. The results obtained in Table 5 and Figure 3 represent the names and classes, in addition to the molecular formula, molecular weight, molecular mass (parent ion M^+^) and base peaks (*m*/*z*) for the eight organic compounds. The main compounds were isophorone and safranal. Also, the extract reactivity elucidated using principal functional groups of IR proved the presence of bands at µ cm^−1^ 3440 cm^−1^ (OH, acid and free), a broad band at 1710–1632 cm^−1^ for α, β-unsaturated carbonyl group, acids and cyclic lactone, a band at 1610–1489 cm^−1^ for (C=C), in addition to a band at 1181 cm^−1^ for ether and cyclic lactone (Figure 4). The main compounds in CSME are isophorone, tetradecanoic acid (acid); 6-hydroxy-4,4,7a-trimethyl-5,6,7,7a-tetrahydrobenzofuran-2(4H)-one (lactone); henyl acetate (ester); 2-naphthalenemethanol, decahydro-à,à,4a-trimethyl-8-methylene (alcohols) and safranal (aldehyde). IR spectroscopy was used to study the principal bands to elucidate the reactivity of the extract towards the biological evaluation screening. 

### 3.6. Transmission Electron Microscopy (TEM) Image Analysis of S. pyogenes Cells Treated with the Antibiotic Imipenem, CSME, BH and CFG

**Figure 5:**
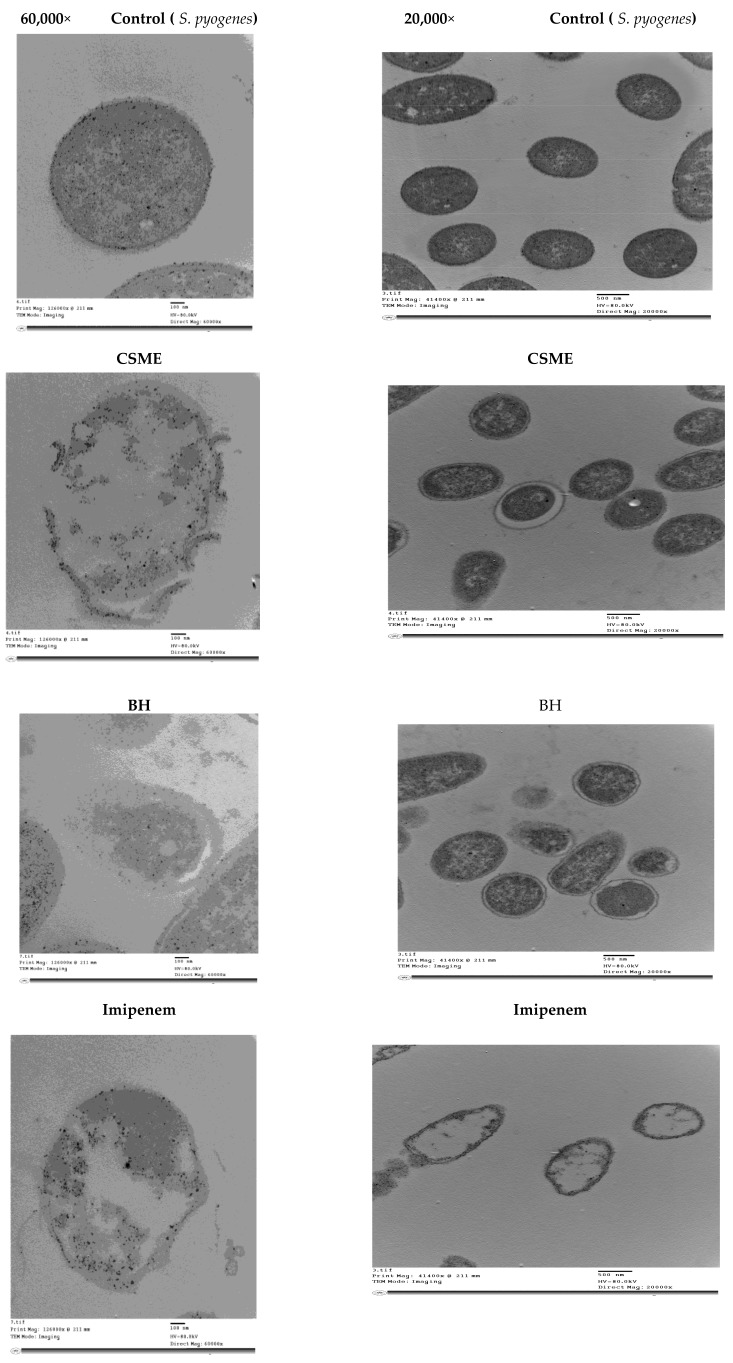

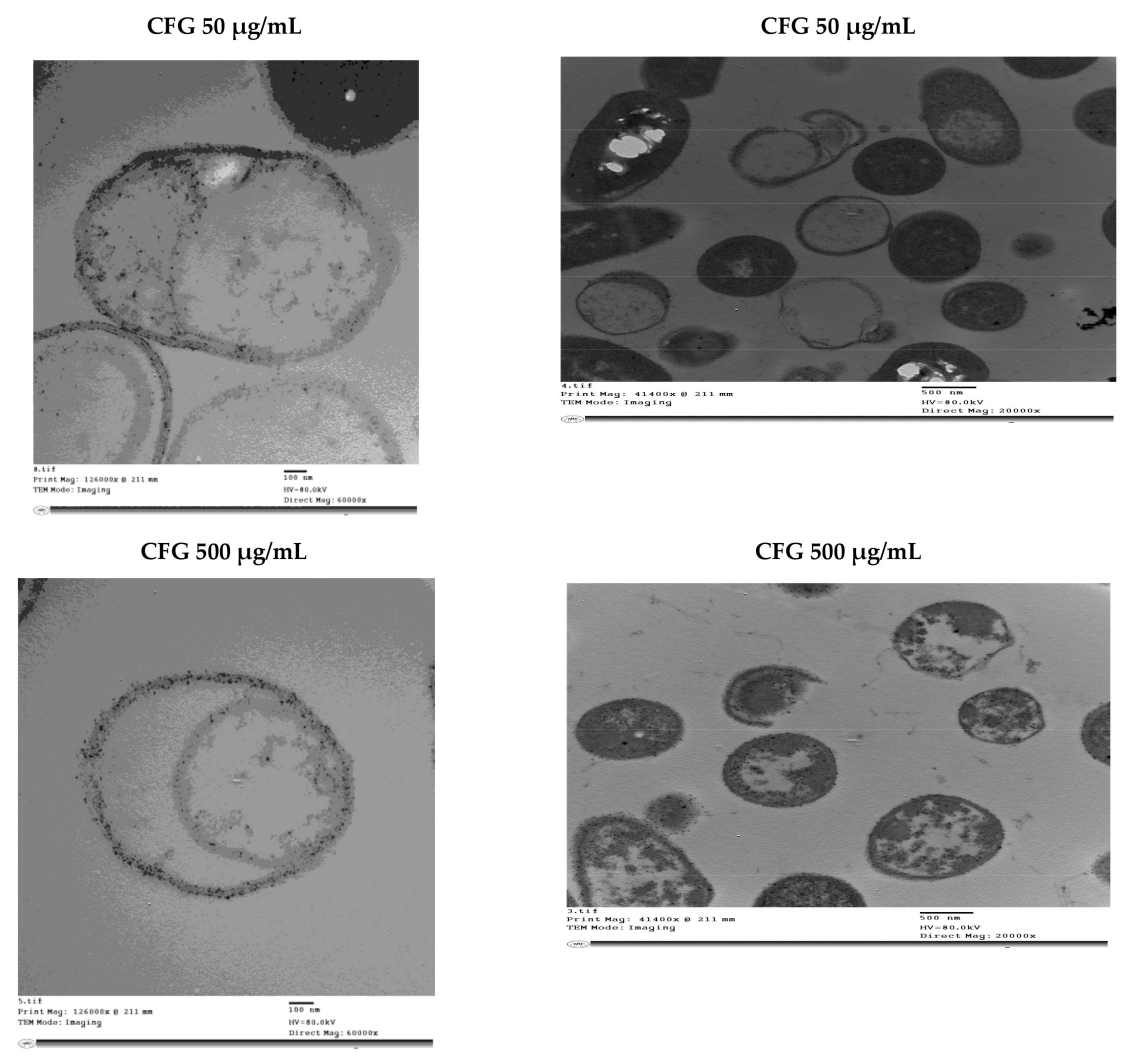
Transmission electron microscopy (TEM) of *S. pyogenes* ZUH1 treated with the MIC of BH, CSME, CFG (50 μg/mL and 500 μg/mL), and imipenem (10 μg/mL) at magnifications of 20,000× and 60,000×.

The most potent anti-streptococcal inhibitors (CSME (20 μg/mL, BH (20%), CFG (50 μg/mL and 500 μg/mL) and imipenem (10 μg/mL)) were mixed with about 2 × 10^5^ CFU/mL of actively growing *S. pyogenes* and examined by TEM. Images showed that the presence of MIC of CSME, BH and 10 μg/mL of imipenem and 50 μg/mL and 500 μg/mL CFG in BHI broth media containing *S. pyogenes* (OD 600 = 0.5 at the time of application) had evidently reduced the relative content of the intact cells after 4 h of incubation at 37 °C (Figure 5). They all induced similar signs including cell shrinkage, cell membrane wrinkling, cell wall lysis, pore formation and emptiness of cellular live material.

## 4. Discussion

*S. pyogenes* is a distinctive bacterial pathogen belonging to Group A streptococci and is responsible for significant morbidity and mortality worldwide. Its infections may spread through direct contact with mucous or sores on the skin and causes >500,000 deaths per year [27]. This shows that there is a need to develop new technologies for the rapid diagnosis of infections caused by GAS. This GAS is implicated in severe pathogenic diseases such as cystitis, pyelonephritis, scarlet fever, sepsis, puerperal fever, urinalysis, pharyngitis, pyodema, cellulitis, necrotizing fasciitis, osteomyelitis, septic arthritis, bacteremia and pneumonia [28,29]. It can also initiate post-infectious diseases such as rheumatic fever and glomerulonephritis. This wide range of infections is another need to continue research into rapid diagnosis of GAS infections and to find out novel treatment protocols for them. This study endeavored to develop rapid molecular diagnosis techniques for GAS and to control the infections using novel treatment protocols based on natural extracts either alone or in combination with effective antibiotics.

The experimental isolate in this study was isolated from a urine swab taken from a 60-year-old man suffering from chronic cystitis. This organism was a Gram positive coccoid and catalase negative. It showed β-hemolytic activity in blood agar and was sensitive to bacitracin. It was ASO positive with an agglutination titer of about 200 IU/mL. All these diagnostic features, in addition to the results obtained from the API-Streptococci Kit, proved that the strain ZUH1 belonged to *Streptococcus pyogenes* (GAS) and thus it was designated as *S. pyogenes* ZUH1 (*S. pyogenes*) [30].

Due to the severity of many infections caused by GAS, rapid diagnosis within 2 h is necessary. Virulence genes are genetic markers and their detection by PCR is a rapid diagnostic test. In this study, the spyCEP gene encodes for a protease that is able to digest the IL-8 protein, resulting in a significant decrease in host phagocytic cell activity. spyCEP was detected in the ZUH1 strain genome, indicating a rapid diagnosis of GAS. This is in agreement with a published letter [4,31]. In addition, the genes speG, sdaB, ssa and sic, encoding superantigens of *S. pyogenes*, were detected in the ZUH1 strain, indicating the rapid detection of GAS [32]

In this study the antibiotic susceptibility profile showed that *S. pyogenes* was sensitive to bacitracin, again indicating that it belonged to the GAS family. Previous published results have shown that the treatment of choice is penicillin, erythromycin, or macrolides and cephalosporins such as clindamycin and amoxicillin/clavulanic acid; however, many reports of antibiotic tolerance have been shown in GAS isolates [33,34,35,36]. In this regard, it is necessary to find out novel technologies to develop other alternative treatment protocols for GAS infections. In this study, CSME and BH were shown to be ideal inhibitory agents for GAS. It was found that the use of biological agents against pathogenic bacteria are needed and could replace chemical agents [37]. Either BH or CSME could be used alone or in combination with different proportions with antibiotics for topical used in the treatment of wound infections caused by GAS. Studies in this respect are still under investigation.

In this study, different organic solvent extracts such as petroleum ether, methanol, benzene, acetone and ethanol could be used to extract the bioactive compounds of *C. sativus*; however methanol was the best for extractability as CSME showed the broadest anti-streptococcal activity and this was similar to previous work in this respect [1,13,38]. BH also inhibited the growth of *S. pyogenes.* The antibacterial nature of BH is dependent on various factors working either alone or synergistically, the most salient of which are H_2_O_2_, phenolic compounds and pH [39]. 

The results of the present investigation clearly indicated that the antimicrobial potential of CFG was similar to previous published results [25]. Most of the reported antimicrobial peptides typically have strong antimicrobial activity against a wide range of Gram-positive and Gram-negative bacteria [11,25]. The results of this study showed that a combination of either CSME or BH with the antibiotic imipenem induced broader anti-streptococcal activity and in turn positive synergistic antibacterial activity against the studied *S. pyogenes* strain, and perhaps such combinations could be promising as a treatment. Thus, evidence of in vitro synergism could be useful in selecting the most favorable combinations of antimicrobials for practical therapy against ZUH1 strain infections. This is similar to that obtained from previous results [9,11]. Studies on the use of CFG as an inhibitory agent for pathogenic bacteria are scarce; therefore, thorough investigation of the biochemical characteristics and antimicrobial activity of CFG are necessary [25]. 

Instrumental analysis such as GC-MS and IR spectroscopy elucidated different bioactive compounds that showed antimicrobial activity such as cyclic ketones, organic acids, aldehydes, alcohols and esters. It was shown that organic acids, aldehydes, and alcohols are fermentative and the products of many probiotics and starter cultures used for fermentations; such metabolic end products decrease the pH of fermented foods so that pathogenic bacteria cannot grow [40]. Isophorones and their derivatives elucidated in this study from CSME showed wide antimicrobial activity against different indicator pathogenic microorganisms such as *Escherichia coli, Bacillus subtilis, Pseudomonas aerugenosa* and *Salomnella typhimurium* [41]. Tetradecanoic acid (myristic acid) is another compound that was found in CSME; it has antifungal, antioxidant, cancer preventive, nematicide, hypercholesterolemic, insecticidal, antibacterial, and anti-inflammatory properties [42]. Safranal is also used as an antimicrobial and anti-tumor agent. Safranal inhibits the growth of *Staphylococcus aureus* [13,15]. Moreover, aldehydes, alcohols and acids are promising antimicrobial agents and used as food biopreservatives [43]. Previous studies showed that benzaldehydes have antibacterial activity against *Listeria monocytogenes, Salomnella enteridis* and *Lactobacillus plantarum* [40]. The anti-streptococcal activity of CSME is due to the mixture of the compounds found from both GC-MS and IR. It is necessary to test the anti-streptococcal activity of each compound alone. Studies in this respect are under investigation. 

TEM images indicated that the natural agents used reduced the content of the intact cells, indicating higher mortality rates. Also, these agents were able to directly disrupt the actions of the cell wall of *S. pyogenes*. This is in agreement with previous studies [11]. 

## 5. Conclusions

The S. pyogenes strain ZUH1 was inhibited by Crocus sativus methanol extract (CSME), bee honey (BH) and catfish glycoprotein (CFG). The inhibitory activity of these natural agents were compared with standard antibiotics such as ceftazidime, cefoperazone, cefoxitin and imipenem. The marker genes spyCEP, ssa, sic, sdaB and speG, indicating group A streptococci (GAS) were detected in the S. pyogenes ZUH1 genome. The synergism between antibiotics and Crocus sativus, bee honey and glycoprotein inhibits the GAS pathogen S. pyogenes ZUH1. The action of natural agents on the pathogenic cells was shown using transmission electron microscopy (TEM). GC-MS analysis of CSME showed different cyclic ketones, aldehydes, esters, alcohols and acids. The main compounds were tetradecanoic acid, safranal and isophorone. 

## Figures and Tables

**Table 1:** Primers used for the detection of the* S. pyogenes* ZUH1 strain virulence factors.

Detected Virulence Factors	Primer Sequence (Forward)	Primer Sequence (Reverse)	Size of the PCR Product (bp)
spyCEP	GATCCGGCCCATCAAAGCAT	AGCTGCCACTGATGTTGGTG	786
ssa	AAGAATACTCGTTGTAGCATGTGT	AATATTGCTCCAGGTGCGGG	678
sic	TTACGTTGCTGATGGTGTATATGGT	TTTGATAGAGGGTTTTCAGCTGGC	150
sdaB	TATAGCGCATGCCGCCTTTT	TGATGGCGCAAGCAAGTACC	440
speG	TGGAAGTCAATTAGCTTATGCAG	GCGAACAACCTCAGAGGGCAAA	384

**Table 2:** Susceptibility of *S. pyogenes* ZUH1 to different antibiotics.

Types of Antibiotic	IZD (mm)
Tetracycline (30 μg/mL)	16.00 e ± 1.5 (S)
Piperacillin (100 μg/mL)	7.00 g ± 0.33 (I)
Clindamycin (2 μg/mL)	10.00 f ± 0.25 (I)
Penicillin G (10 μg/mL)	19.00 d ± 0.41 (S)
Ceftazidime (30 μg/mL)	25.00 b ± 0.11 (S)
Cefoxitin (30 μg/mL)	24.00 b ± 0.65 (S)
Cefoperazone (75 μg/mL)	24.00 b ± 0.13 (S)
Imipenem (10 μg/mL)	36.00 a ± 0.15 (S)
Amikacin (30 μg/mL)	21.00 c ± 0.26 (S)
Ampicillin (10 μg/mL)	R
Aztreonam (10 μg/mL)	R
Norfloxacin (10 μg/mL)	R

R = resistant; S = sensitive; I = intermediate; IZD = Inhibition zone diameter. Means in the same column with different letters are significantly different (*p * ≤ 0.05).

**Table 3:** Antibacterial activity of different concentrations of *Crocus sativus* methanol extract (CSME), bee honey (BH) and catfish glycoprotein (CFG) against *S. pyogenes* ZUH1.

Concentrations of CSME (µg/mL)	0	10	20	30	40	50	60	70	80	90
IZD (mm)	0.00 g	0.00 g	5.00 f ± 0.25	10.00 e ± 0.22	12.00 e ± 0.15	15.00 d ± 0.16	18.00 c ± 0.45	24.00 b ± 0.36	26.00 b ± 0.75	32.00 a ± 0.11
**Concentrations of BH (g/100 mL)**	10	20	30	40	50	60	70	80	90	100
IZD (mm)	0.00 f	8.00 e ±0.35	15.00 d ± 0.25	18.00 d ± 0.29	25.00 c ± 0.71	26.00 c ± 0.11	28.00 bc ± 0.23	30.00 b ± 0.43	36.00 a ±0.13	38.00 a ± 0.43
**Concentrations of CFG (µg/mL)**	0	10	25	50	100	250	500			
IZD (mm)	0.00 d	0.00 d	0.00 d	19.00 c ±0.13	24.00 b ±0.45	30.00 a ±0.34	32.00 a ± 0.65			

Means in the same row with different letters are significantly different (*p * ≤ 0.05).

**Table 4:** Antibacterial activity of mixed combinations of different antibiotics and either CSME or BH against *S. pyogenes*.

Antibiotic	IZD of Antibiotics Only	IZD of Antibiotics with BH *	IZD of Antibiotic with CSME **
Ceftazidime (30 μg/mL)	26.00 b ± 0.25	35.00 c ± 0.11	30.00 c ± 0.16
Cefoperazone (75 μg/mL)	25.00 b ± 0.23	34.00 c ± 0.35	32.00 b ± 0.76
Cefoxitin (30 μg/mL)	25.00 b ± 0.34	38.00 b ± 0.54	33.00 b ± 0.24
Imipenem (10 μg/mL)	34.00 a ± 0.23	42.00 a ± 0.51	36.00 a ± 0.55

IZD: inhibition zone diameter; * BH was added at 20 g/100 mL; ** CSME was added at 20 μg/mL. Means in the same column with different letters are significantly different (*p *≤ 0.05).

**Table 5:** The structure, molecular formula and molecular weight (M.W.) of 8 compounds from *Crocus sativus* methanol extract (CSME) when subjected to GC-MS (gas liquid chromatographic mass spectrometry).

No.	Compounds Name	M.W.	Molecular Formula	M^.+^ Parent Ion	Area	Base Peak	Classification
1	Isophorone	138.0	C_9_H_14_O	138.0	2.50	29.0	Cyclic ketone
2	3-Hydroxy-7,8-dihydro-á-1-ionol	208.0	C_13_H_20_O2	209.0 (M^+1^)	1.24	43.0	Alcohol
3	2-Naphthalenemethanol, decahydro-à,à,4a-trimethyl-8-methylene	222.0	C_15_H_26_O	222.0	4.25	59.0	Alcohol
4	Phenyl acetate	338	C_22_H_42_O	338.0	4.25	43.0	Ester
5	Tetradecanoic acid	228.0	C_14_H_28_O_2_	228.0	6.65	73.0	Acid
6	1,3-Cyclohexadiene-1-carboxaldehyde, 2,6,6-trimethyl-(Safranal)	150	C_10_H_14_O	150	23.12	39.0	Aldehyde
7	6-Hydroxy-4,4,7a-trimethyl-5,6,7,7a-tetrahydrobenzofuran-2 (4H)-one	196.0	C_11_H_16_O_3_	196.0	5.12	111.0&43.0	Lactone (cyclic esters)
8	2,5,7,8-Tetramethyl-2-(4,8,12-Trimethyltridecyl)-6-chromanol	430.0	C_29_H_50_O_2_	430.0	3.12	165.0	Natural product (cyclic ether)

## Supplementary Materials

The following are available online at https://www.mdpi.com/1420-3049/24/16/2903/s1, Figures S1–S4.
