# Supplementary material for: Biological Characterization and Inhibition of Streptococcus pyogenes ZUH1 Causing Chronic Cystitis by Crocus sativus Methanol Extract, Bee Honey Alone or in Combination with Antibiotics: An In Vitro Study
Source: Molecules. 2019 Aug 9;24(16):2903. doi: 10.3390/molecules24162903 (PMC6721159; doi:10.3390/molecules24162903)
Supplement: Supplementary file 1 [file molecules-24-02903-s001.pdf]

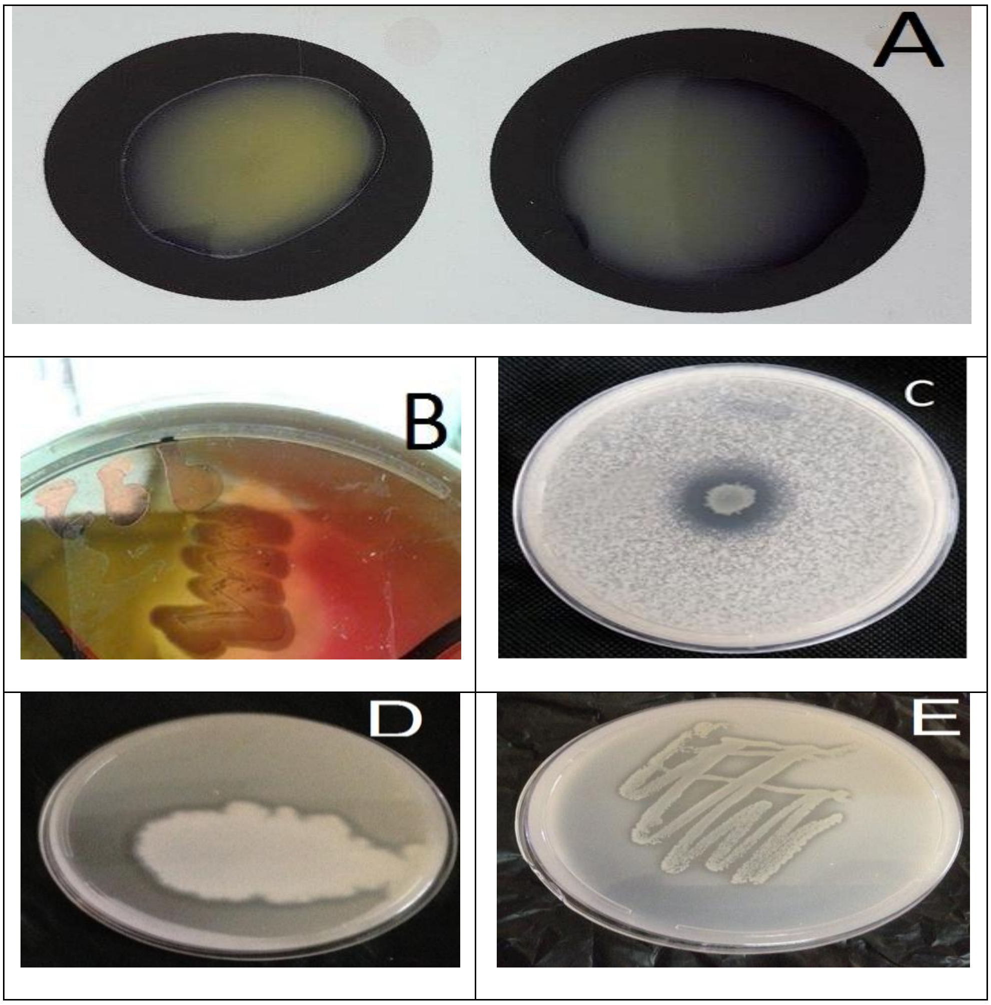

Supplementary Figure 1. Biochemical characteristics of *S. pyogenes* ZUH1 (A): antistreptolysin O assay, (B): blood hemolysis test,(C):Lipase activity, (D): Phospholipase activity and (E): Protease activity.

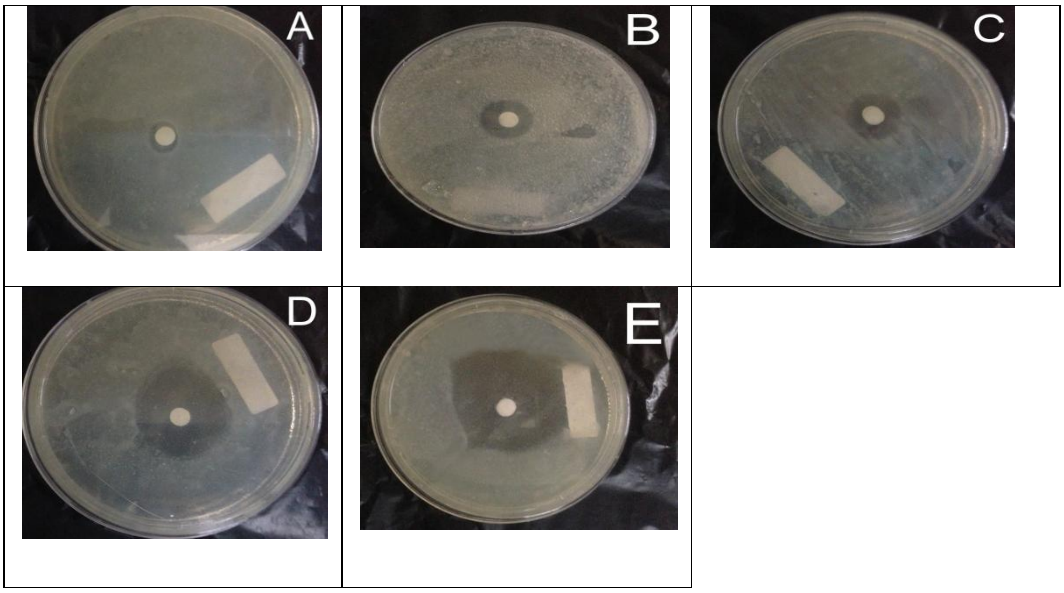

Supplementary Figure 2. Antibacterial activity of *C. sativus* with (A): ethanol extract,(B): benzene extract, (C): petroleum ether extract,(D): acetone extract, (E): methanol extract against *S. pyogenes*

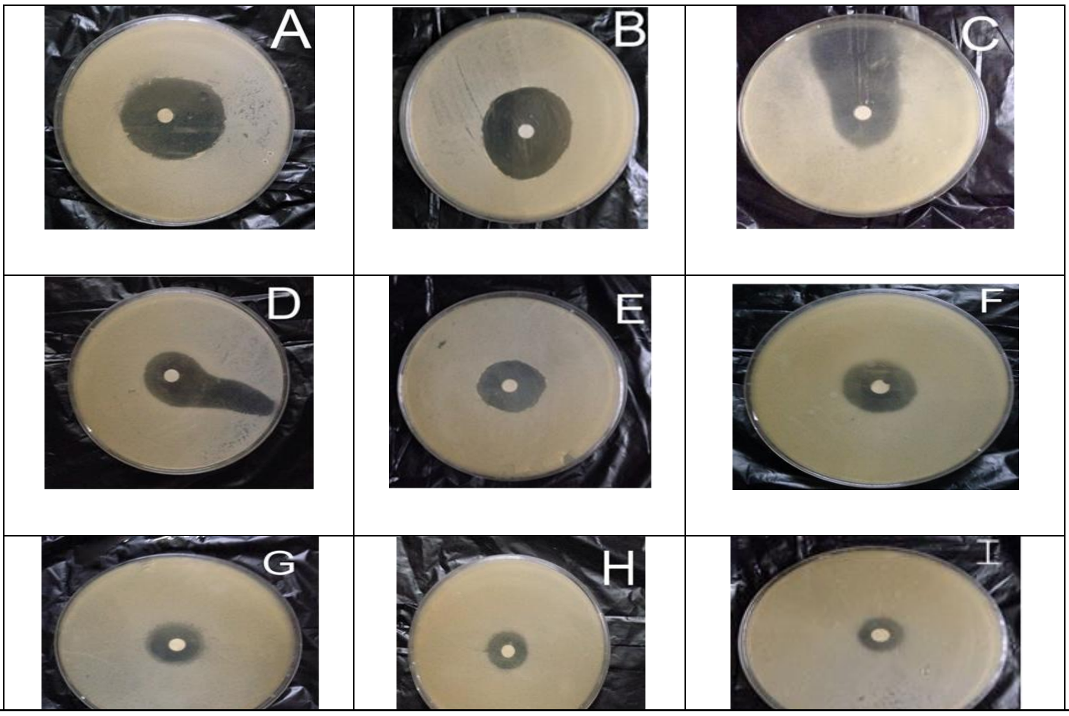

Supplementary Figure 3. **Antibacterial activity using disc diffusion assay against *S. pyogenes* ZUH1 of (A): Crude BH (100 %), (B): 90 % concentration, (C): 80 % concentration, (D): 70 % concentration, (E): 60 % concentration, (F): 50 % concentration, (G): 40 % concentration, (H): 30 % concentration, (I): 20 % concentration.**

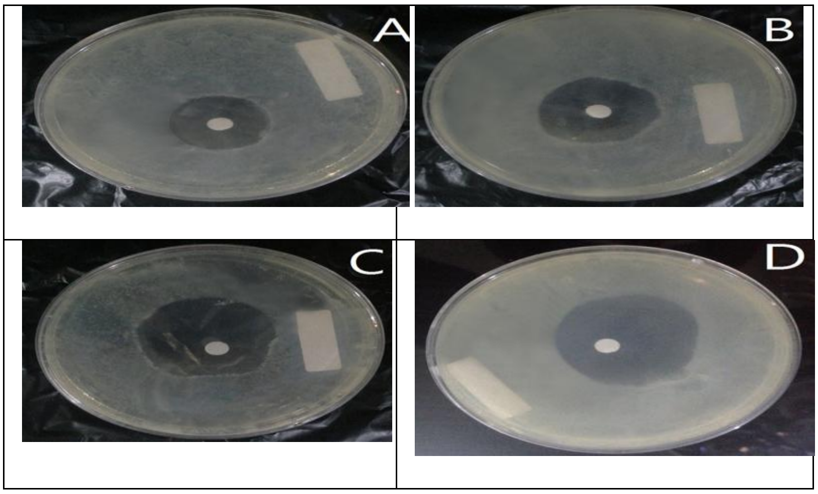

Supplementary Figure (4): Antibacterial activity of CFG against *S. pyogenes* ZUH1, (A): 50 μg/mL (B): 100 μg/mL (C): 250 μg/mL (D): 500 μg/mL.
